# A Novel Class of Anti-HIV Agents with Multiple Copies of Enfuvirtide Enhances Inhibition of Viral Replication and Cellular Transmission In Vitro

**DOI:** 10.1371/journal.pone.0041235

**Published:** 2012-07-23

**Authors:** Chien-Hsing Chang, Jorma Hinkula, Meiyu Loo, Tina Falkeborn, Rongxiu Li, Thomas M. Cardillo, Edmund A. Rossi, David M. Goldenberg, Britta Wahren

**Affiliations:** 1 Immunomedics, Inc., Morris Plains, New Jersey, United States of America; 2 IBC Pharmaceuticals, Inc., Morris Plains, New Jersey, United States of America; 3 Department of Microbiology and Tumor Biology, Karolinska Institutet, Stockholm, Sweden; 4 Department of Molecular Virology, Linkoping University, Linkoping, Sweden; 5 Center for Molecular Medicine and Immunology, Garden State Cancer Center, Morris Plains, New Jersey, United States of America; Pasteur Institute of Shanghai, Chinese Academy of Science, China

## Abstract

We constructed novel HIV-1 fusion inhibitors that may overcome the current limitations of enfuvirtide, the first such therapeutic in this class. The three prototypes generated by the Dock-and-Lock (DNL) technology to comprise four copies of enfuvirtide tethered site-specifically to the Fc end of different humanized monoclonal antibodies potently neutralize primary isolates (both R5-tropic and X4-tropic), as well as T-cell-adapted strains of HIV-1 in vitro. All three prototypes show EC_50_ values in the subnanomolar range, which are 10- to 100-fold lower than enfuvirtide and attainable whether or not the constitutive antibody targets HIV-1. The potential of such conjugates to purge latently infected cells was also demonstrated in a cell-to-cell viral inhibition assay by measuring their efficacy to inhibit the spread of HIV-1_LAI_ from infected human peripheral blood mononuclear cells to Jurkat T cells over a period of 30 days following viral activation with 100 nM SAHA (suberoylanilide hydroxamic acid). The IgG-like half-life was not significantly different from that of the parental antibody, as shown by the mean serum concentration of one prototype in mice at 72 h. These encouraging results provide a rationale to develop further novel anti-HIV agents by coupling additional antibodies of interest with alternative HIV-inhibitors via recombinantly-produced, self-assembling, modules.

## Introduction

There are about 32 antiretroviral products approved for the treatment of the HIV-1/AIDS pandemic [1], with 26 formulated singly and 6 in combination, in 7 different classes: nucleoside reverse transcriptase inhibitors (NRTIs), non-nucleoside reverse transcriptase inhibitors (NNRTIs), protease inhibitors (PIs), fusion inhibitors, entry inhibitors, HIV integrase strand transfer inhibitors, and multi-class combination products. Although the use of highly active antiretroviral therapy (HAART), which comprises two, three or more anti-HIV-1 drugs selected from NRTIs, NNRTIs, and PIs, has improved the prognosis for individuals infected with HIV-1 significantly, and can reduce plasma viral loads below the detection limits (50 copies HIV RNA/mL) of standard clinical assays, a cure remains elusive. Thus, there is a need for new anti-HIV agents or approaches, with the ultimate challenge of eradicating latent HIV-1 reservoirs [2], [3], particularly when considering the lifelong requirement of HAART to control the rebound of latent or persistently replicating virus, the toxicities associated with long-term treatment, and the growing concerns for the side-effects and cost of such chronic therapies.

Enfuvirtide (called T20 herein) was the first drug in the class of HIV-1 fusion inhibitors to receive approval in 2003 for treating AIDS patients [4], [5]. We envisioned a novel class of anti-HIV agents having multiple copies of T20 stably tethered onto an antibody of choice. Such agents can be conveniently generated by the Dock-and-Lock (DNL) platform technology [6] to comprise four copies of T20 linked to an IgG. Collectively termed IgG-(T20)_4_, they are expected to provide the therapeutic benefits of T20 with the added advantages conferred by the IgG component, one of which would be improved pharmacokinetics with a longer serum half-life to allow less frequent dosing than the twice daily currently required for T20. Moreover, depending on the targeting specificity and effector functions of the conjugated antibody, whether binding, neutralizing or not, the resulting DNL constructs could eliminate both infected cells and free virus via several known mechanisms [7]–[9], including complement-mediated lysis, antibody-dependent cellular cytotoxicity (ADCC), antibody-dependent cell-mediated virus inhibition (ADCVI), and induction of apoptosis.

Among the various antibodies that bind and neutralize HIV-1, the murine anti-gp120 (V3 loop) antibody, P4/D10, is distinguished by its additional feature of inducing ADCC to eliminate infected T cells [10]. Enhanced potency was also observed for a doxorubicin-conjugated P4/D10 to neutralize free virus and inhibit intercellular spread of viral infection in vitro, as well as to protect against HIV-1/MuLV infection in a murine model [11]. To reduce its potential immunogenicity, we have constructed a human-mouse chimeric P4/D10 (cP4/D10) and demonstrated it is as effective as the parental P4/D10 in neutralizing HIV-1 in vitro. We have now also generated the IgG-(T20)_4_ of cP4/D10 and two humanized mAbs, namely, h734, a non-immunoreactive variant of *h*734 (anti-indium-DTPA IgG_1_), and hLL2 or epratuzumab (anti-CD22 IgG_1_), that do not bind to HIV-1, for assessing the effect of other cellular targeting on anti-HIV potency. Our results show, surprisingly, that each of the three DNL conjugates of T20, designated cP4/D10-(T20)_4_, h734-(T20)_4_, and hLL2-(T20)_4_, is more potent than T20 in inhibiting HIV-1 in vitro, even when the antibody has no HIV-1-specificity, and that the hLL2-(T20)_4_ conjugate clears from blood in mice in a prolonged time, similar to unconjugated hLL2 IgG.

## Results

### Development of IgG-(T20)_4_

**Figure 1:**
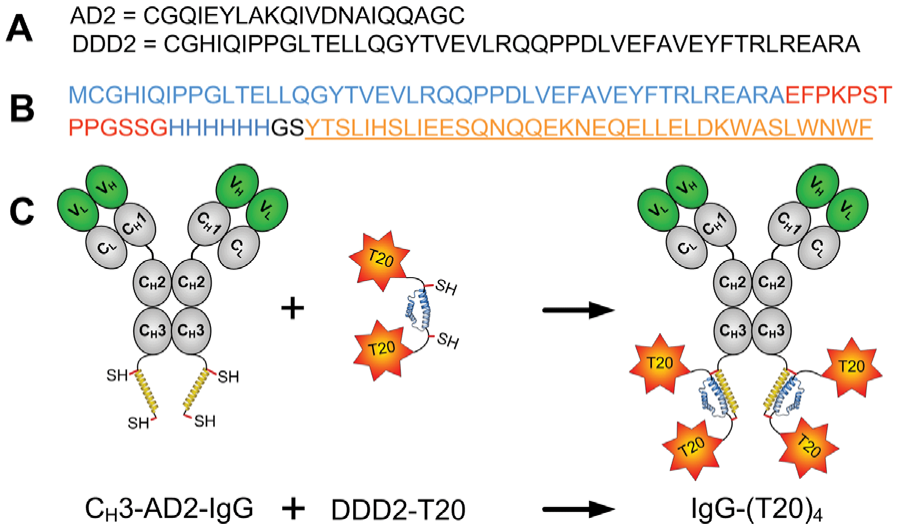
Design and construction of IgG-(T20)_4_, showing (A) amino acid sequences of AD2 and DDD2, (B) amino acid sequence of DDD2-T20, and (C) conjugation of IgG-AD2 with DDD2-T20 to generate IgG-(T20)_4_.

The basic strategy of DNL involves the construction of two types of modules, one containing the dimerization and docking domain (DDD) of cAMP-dependent protein kinase A (PKA), referred to as the DDD module, and the other bearing the anchoring domain (AD) of an interactive A-kinase anchoring protein (AKAP), referred to as the AD module. Among the distinctive features of DNL are the spontaneous formation of a dimer of the DDD module and the self-assembly of the DDD module with the AD module into a non-covalent complex, which is subsequently rendered covalent with disulfide bonds to enhance stability in vivo [12]. The amino acid sequences of a pair of DDD and AD linkers useful for the DNL conjugation (termed DDD2 and AD2, respectively) are shown in Fig. 1A. We engineered the DDD2 sequence to the N-terminus of T20 via a flexible peptide of 21 amino acid residues, which also contains a 6xHis-tag to facilitate purification by immobilized metal ion affinity chromatography. The resulting fusion protein, termed DDD2-T20 (Fig. 1B), was expressed in *E. coli*, and isolated from the cell lysates by HIS-Select without refolding and further purification. Although the DDD2-T20 was found to comprise a mixture of crosslinked congeners of varying molecular sizes, the identity of DDD2-T20 was verified by LC-MS (Fig. S1), which showed the fully reduced form of DDD2-T20 to have the mass predicted for the monomeric composition (11824.92, found; 11825, expected). Various batches of DDD2-T20 with similar qualities have been prepared and used to produce IgG-(T20)_4_ by combining with an available IgG-AD2 of interest, as illustrated in the schematic diagram shown in Fig. 1C. Representative SE-HPLC chromatograms of two IgG-AD2 modules (hLL2-IgG-AD2 and cP4/D10-IgG-AD2) and their respective DNL complexes with DDD2-T20 are shown in Fig. S2. The generations of cP4/D10 and cP4/D10-IgG-AD2 are described in Methods S1. P4/D10 and cP4/D10 exhibited similar binding to both the HIV-1 gp160 envelope protein (Fig. S3A) and the V3-peptide epitope (Fig. S3B).

### In vitro neutralization of cell-free HIV-1 by IgG-(T20)_4_

**Figure 2:**
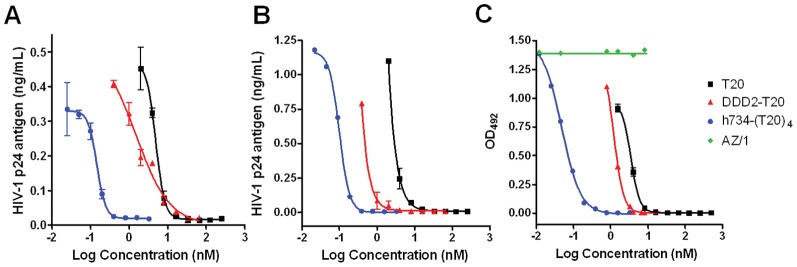
Inhibition of HIV-1_6920_ replication in PBMCs by h734-(T20)_4_, DDD2-T20 and T20 as determined by p24 antigen ELISA at day 9 (A) or day 16 (B). The inhibition of cell-to-cell infection of HIV-I_IIIB_ in Jurkat T cells (2% HIV infected cells mixed with 98% uninfected cells) is shown in (C). The control mAb (AZ/1) with specificity for human cytomegalovirus protein p65 shows no effect.

The activities of DDD2-T20, h734-(T20)_4_, and the unconjugated T20 to inhibit the infection of HIV-1_6920_, a primary, nonsyncytium-inducing (NSI), CCR5-tropic, clade B isolate [13], to human peripheral blood mononuclear cells (PBMCs) were determined by HIV p24 ELISA [14] and compared in Fig. 2A–C. Because h734-(T20)_4_ is significantly higher in molecular weight (∼200,000 Da) than either DDD2-T20 (11,825 Da as monomer) or T20 (4,492 Da), these data are presented in molar concentrations. Both a standard 9-day neutralization assay (Fig. 2A) and a prolonged 16-day assay (Fig. 2B) reveal the superior potency of h734-(T20)_4_ over both DDD2-T20 and T20. The h734-(T20)_4_ complex exhibited an EC_50_ of about 0.1 nM, compared to about 2 nM for DDD2-T20 (calculated as the monomer), and about 10 nM for T20. Similar results showing the higher potency of h734-(T20)4 compared to DDD2-T20 and T20 were also observed (Fig. 2C) in a cell-to-cell inhibition assay of HIV-1IIIB [15], [16] in Jurkat T cells, where AZ/1, a control IgG directed against cytomegalovirus protein gp65, exhibited no activity.

**Figure 3:**
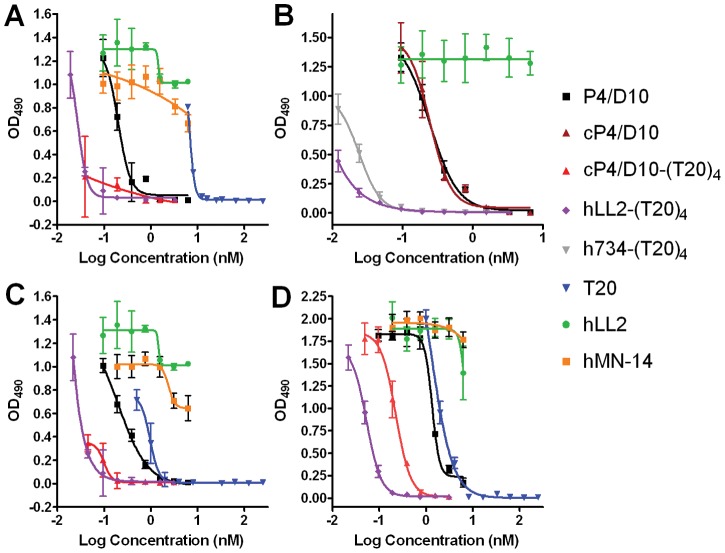
Comparing the potency of P4/D10, cP4/D10, h734-(T20)_4_, and hLL2-(T20)_4_ for neutralization of HIV-1_IIIB_ in Jurkat T cells dosed at 50 TCID_50_ (A) and 100 TCID_50_ (B), and HIV-1_6794_ in PBMCs dosed at 50 TCID_50_ (C) and 100 TCID_50_ (D).

The enhanced in vitro efficacy of the IgG-(T20)_4_ constructs to inhibit viral replication was further attested by comparing the potency of P4/D10, cP4/D10, cP4/D10-(T20)_4_, h734-(T20)_4_, and hLL2-(T20)_4_ for neutralization of HIV-1_IIIB_ in Jurkat T cells (Fig. 3A, B) and HIV-1_6794_, a primary, syncytium-inducing (SI), CXCR4-tropic, clade B isolate [17], in PBMCs, with the results indicating that (i) cP4/D10 and P4/D10 are equivalent in their potency to neutralize HIV-1_IIIB_ in Jurkat T cells (Fig. 3B); (ii) cP4/D10-(T20)_4_ is more potent than P4/D10 in neutralizing HIV-1_1IIIB_ (Fig. 3A) and HIV-1_6794_ (Figs. 3C,D); (iii) both hLL2-(T20)_4_ and h734-(T20)_4_ are as potent as cP4/D10-(T20)_4_ in neutralizing HIV-1 (Figs. 3B–D); and (iv) neither of the control antibodies, hLL2 or hMN-14 (anti-CEACAM5), has neutralization activity. Table 1 summarizes the EC_50_ values for HIV neutralization estimated from the results shown in Figs. 2A and 3A–D and additional experiments performed in parallel.

**Table 1:** Estimated EC_50_ (nM)^a^.

	HIV-1_6920_ (R5-tropic)		HIV-1_IIIB_ (X4-tropic)		HIV-I_6794_ (X4-tropic)	
	Jurkat T	PBMC	Jurkat T		PBMC	
			50TCID_50_	100TCID_50_	50TCID_50_	100TCID_50_
T20	3	10	5	1	3	1
P4/D10	8	25	0.5	0.4	0.3	2
cP4/D10	10	30	0.6	0.4	0.85	0.95
cP4/D10-(T20)_4_	<0.04	<0.04	<0.04	<0.02	<0.04	0.2
h734-(T20)_4_	0.07	0.1	ND	0.02	ND	ND
hLL2-(T20)_4_	ND	<0.07	0.03	<0.01	0.02	0.1

a Data compiled from Fig. 2, Fig. 3, and from additional experiments performed in parallel.

ND, not determined.

### Inhibition of cell-to-cell transfer of virus after SAHA activation

**Figure 4:**
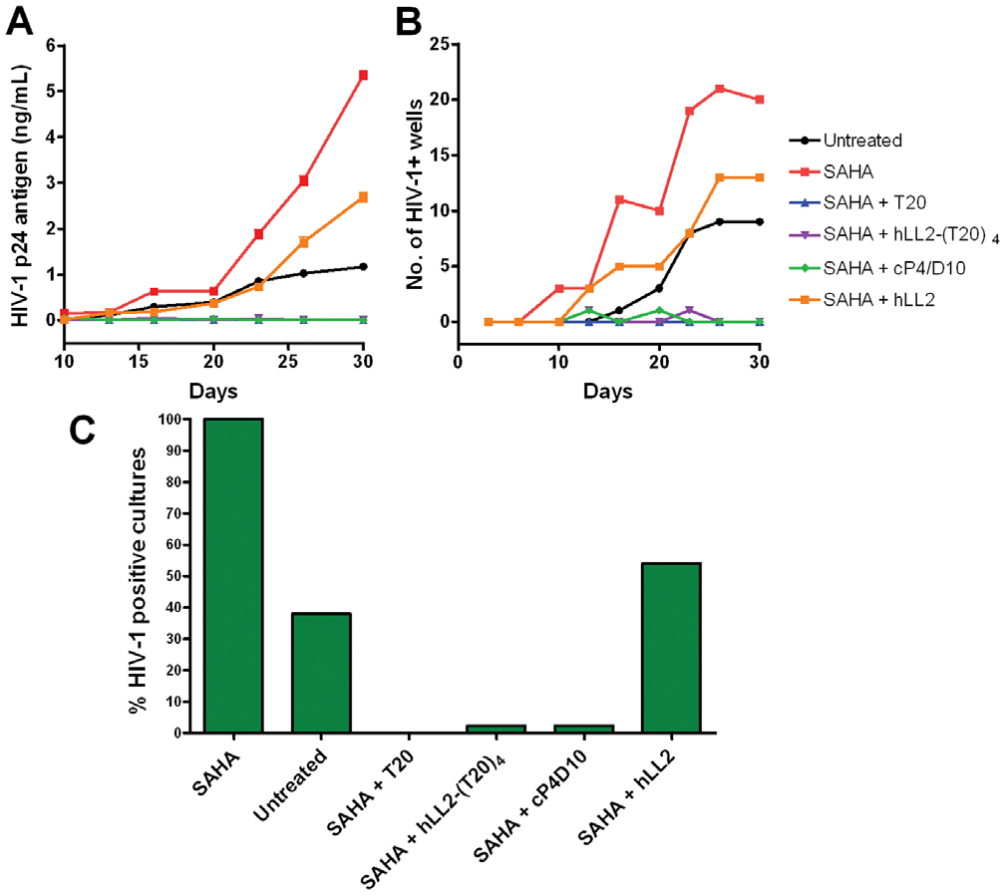
Neutralization of HIV-1_LAI_ in PBMCs following activation of latent virus by SAHA over a period of 30 days as monitored by p24 antigen (A) and numbers of HIV-positive cultures (B). The virus-positive cultures on Day 30 in cells treated with each agent are shown in (C) as a percent of the medium with SAHA-treated control.

The potential of IgG-(T20)_4_ to purge latently infected cells was investigated in a cell-to-cell viral inhibition assay by measuring the efficacy of hLL2-(T20)_4_ to inhibit the spread of HIV-1_LAI_ from infected PBMCs to Jurkat T cells over a period of 30 days following activation with 100 nM SAHA (suberoylanilide hydroxamic acid). For comparison, cP4/D10, T20, and hLL2 also were included. A substantial and persistent increase in HIV replication, measured by either p24 ELISA (Fig. 4A) or p24-positive cultures (Fig. 4B), was observed in SAHA-added medium throughout the 30-day period, which could be nearly or completely suppressed by cP4/D10, T20 or hLL2-(T20)_4_. On day 30, as shown in Fig. 4C, each of the three agents reduced p24-positive cultures to less than 5% of the medium+SAHA control.

### In vivo stability of IgG-(T20)_4_ in mice

**Figure 5:**
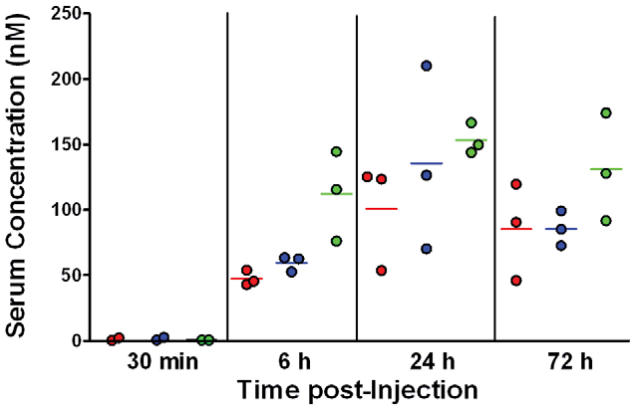
Concentrations of intact hLL2-(T20)_4_ (•) and all hLL2-containing species (•) in serum samples collected from mice at 30-min, 6-h, 24-h, and 72-h, post-injection of hLL2-T20, compared with concentrations of hLL2 (•) in serum samples collected from mice at the same time points post-injection of hLL2.

As shown in Fig. 5, hLL2-(T20)_4_ appears to be stable in vivo for at least 3 days, because the serum concentrations measured by the two assays, one designed to quantify only the intact hLL2-(T20)_4_ and the other to determine all the hLL2-containing species, were comparable at 6, 24 and 72 h. Based on these results, the bioavailability, as determined by the mean serum concentration of hLL2-(T20)_4_ at 72 h, was no less than half that of hLL2 (85.3±37.2 vs. 131.2±41.3 nM), with the difference not being statistically significant (*P* = 0.2263, two-tailed *t*-test).

## Discussion

The DNL method combines genetic engineering with site-specific conjugation to enable self-assembly of two modular components only with each other, resulting in a covalent structure of defined composition with retained bioactivity [6]. To date, we have successfully applied this technology to generate various types of multivalent, multispecific structures that encompass mono- and bi-specific hexavalent antibodies [18], [19], mono-PEGylated dimeric interferon-α2b [20], and tetrameric interferon-α2b linked to a targeting antibody [21], all of which were designed to have the properties desirable for a therapeutic agent, such as target specificity, prolonged serum half-life, higher binding avidity, and enhanced potency. Because the DNL approach allows facile generation of prospective candidates through the modular combination of different antibodies, in particular, broadly binding or neutralizing anti-HIV antibodies [22], and other HIV-fusion or entry inhibitors [23], the proof-of-concept demonstrated herein could lead to many other anti-HIV agents by further evaluation of additional antibodies of interest and alternative HIV-inhibitors as components of the DNL conjugates.

Besides cP4/D10, which serves as a representative of neutralizing anti-HIV antibodies with ADCC, the prospect of enhancing the potency of T20 via conjugation to antibodies not targeting HIV was investigated and demonstrated in vitro with h734 and hLL2. Thus, even for a non-targeting antibody, its IgG-(T20)_4_ derivative may acquire a substantially higher activity in inhibiting or neutralizing the infectivity of HIV-1 than unconjugated T20, which is a notable advantage over certain conjugates of fusion inhibitors not made by DNL (Table S1). For example, EP40111 [24], a PEG-pentasaccharide conjugate of T20, had a substantially decreased potency compared to unconjugated T20; and PC-1505 [25], a human serum albumin conjugate of C34 (a T20 analog that shows somewhat improved efficacy, but similar half-life compared to T20), did not show an improved potency over T20. Taken together, these results suggest, unexpectedly, that the efficacy of an HIV-fusion inhibitor in general, and T20 in particular, may be improved by incorporating it as multiple copies into a DNL complex with a wide variety of antibodies that are neither neutralizing nor directed against the cell-surface receptor (CD4) or coreceptors (CCR5 and CXCR4) of HIV. However, the mechanism of such enhanced potency is not readily apparent at present.

On the other hand, newer antibodies with broader and more potent HIV-binding and neutralization activities across clades are continuously being identified or engineered [26], as exemplified by the more recently reported VRC01 [27], NIH-45-46 [28], and PGT128 [29]. It is conceivable that these broader neutralizing antibodies, as well as selective CD4-, CXCR4-, or CCR5-targeting antibodies that block the entry of HIV-1, may be either used as the IgG components of the DNL conjugates to expand the future repertoire, or co-administered with a T20-containing DNL complex to achieve a further increase in efficacy. Likewise, replacing T20 with either next-generation fusion inhibitors, particularly those showing potent activity against enfuvirtide-resistant virus [30], or RANTES-based entry inhibitors [31], [32], may confer additional benefits.

Current strategies designed to enhance anti-HIV potency via simultaneously intervening multiple aspects of the HIV-1 entry process have evolved from the earlier combination of gp41-targeting T20 with either the CD4-attachment inhibitor PRO 542 [33], or the CD4-binding humanized antibody, TNX-355 [34], demonstrating synergistic antiviral activity in vitro, to the latest development of novel chimeric inhibitors generated by covalently linking C37 (a C-peptide of gp41 fusion inhibitor) to griffithsin [35], which binds to the carbohydrates of gp120/gp41 and inactivates HIV-1 [36], or C37 to a CCR5-targeting RANTES variant [37] highly potent in inhibiting the entry of R5-tropic virus [31]. Alternative strategies to enhance anti-HIV potency via increasing the apparent affinity for HIV-1-binding antibodies have also proved to be successful with the dimeric form of the domain-swapped, anti-gp120/carbohydrate 2G12 [38], [39], and an engineered bispecific antibody that colligates both gp120 and gp41 subunits of the gp160 viral surface spike [40]. All of these strategies, as well as existing approaches to exploit immunotoxins [41], immunodrugs [11], [42], radioimmunoconjugates [43], PEGylated interferon-α [44], and targeted delivery of siRNAs with scFv [45] or aptamer [46], as improved anti-HIV agents, are adaptable with the DNL technology by developing relevant modules [47].

The three prototypes of IgG-(T20)_4_ studied are highly active against primary isolates (both R5-tropic and X4-tropic) and T-cell-adapted strains of HIV-1, showing inhibitory EC_50_ values in the subnanomolar range, which are 10- to 100-fold more potent than enfuvirtide (Table 1) and, remarkably, can be attained with both HIV-targeting (cP4/D10) and non-targeting antibodies (h734 and hLL2). These in vitro antiviral results are comparable in efficacy to those reported for recombinant proteins comprising next-generation fusion inhibitor peptides attached to the Fc end of an entry-blocking antibody targeting either CCR5 [48] or CD4 [49]. The CCR5-targeting fusion protein, however, was not effective against the replication of X4- or R5X4-tropic viruses in PBMCs [48], in which only a small subset of CD4- T cells express CCR5, while most CD4- T cells express CXCR4 [50], [51]. It remains to be determined whether the improved potency of hLL2-(T20)_4_ or h734-(T20)_4_ results from increased binding of the clustered T20 to the exposed gp41 or Fc receptor-mediated antiviral activities [7], or both. Indeed, targeting by the cP4D10 anti-HIV antibody compared to non-HIV IgG may not have been potent enough to override the efficacy evoked by a pair of dimers of T20 in proximity.

The current production process of T20 is complex, requiring numerous synthetic steps. Thus, the feasibility to produce functional DDD2-T20 in *E. coli* is a notable advance and should have a substantial reduction on manufacturing costs for future T20-derived therapeutics. Moreover, the generation of IgG-(T20)_4_ is relatively simple, which we have accomplished with several other types of IgG-based DNL conjugates, including IgG linked to interferon-α, interferon-λ1, ranpirnase, G-CSF, EPO, and human protamine. Based on the current data of in vitro potency and IgG-like half-life, we do not expect a large dose of IgG-(T20)_4_ would be needed either.

Before entering human trials, key preclinical studies will include in vitro testing of efficacy in inhibiting enfuvirtide-resistant virus, and in vivo evaluations in appropriate murine and primate models to assess biodistribution, pharmacokinetics, immunogenicity, and dose-response effects. We envision the primary target AIDS patient population for IgG-(T20)_4_ to comprise individuals failing HAART therapy, where several doses of the DNL conjugates may effectively reduce the number of infected cells and circulating virions. Another potential patient population comprises individuals on effective HAART, with the goal of targeting and deleting the few persisting, viral-antigen-exposing or producing, cells. Any potential immunogenicity of these engineered constructs should be mitigated by use of humanized or chimeric antibodies, especially when administered to immunosuppressed AIDS patients.

## Materials and Methods

### Strains, cells, and reagents

Jurkat T cells were originally purchased from ATCC (Manassas, VA) by The Swedish Institute for Communicable Disease Control. Jurkat T-cell line-adapted strains (HIV-1_IIIB_ and HIV-1_Lai_), primary isolates (HIV-1_6920_ and HIV-1_6794_), all of clade B, and, were obtained from The Swedish Institute for Communicable Disease Control. The phenotypes and biological properties of HIV-1_6920_ and HIV-1_6794_ have been described [13], [17]. The collection and usage of human blood samples, with signed informed consent, was approved by the Local Ethical Committee of Karolinska Institutet (Stockholm, Sweden) and the Local Ethical Committee of Linköping City University Hospital (Linköping, Sweden). PBMCs were isolated by Ficoll-Hypaque density-gradient centrifugation from whole blood collected from healthy donors in EDTA-tubes/bags. The HIV-1 p24 antigen capture ELISA was performed as described previously [14]. Descriptions of the chimerization of P4/D10; the construction of expression vectors, protein production and purification for cP4/D10, cP4/D10-AD2 and T20-DDD2; and, conjugation (DNL) and purification of IgG-(T20)_4_ constructs are provided in Methods S1.

### In vitro neutralization assays

Virus neutralization was performed against HIV-1_IIIB_ and HIV-1_6794_ (or HIV-1_6920_) in Jurkat T cells and PHA/IL-2 activated PBMCs, respectively. Briefly, HIV-1 viruses (50 or 100 TCID_50_) were mixed in duplicate with serially diluted test articles in complete medium (RPMI 1640 supplemented with 10% inactivated FCS, 10 IU/mL IL-2, 50 nM 2-mercaptoethanol, 4 mM L-glutamine, 2 mM sodium pyruvate, and 1% gentamicine) in a final volume of 100 µL/well. After incubation for 1 h (37°C, 5% CO_2_), Jurkat T cells (50,000 cells/well) or PBMCs (150,000 cells/well) were added (100 µL/well) and incubated overnight (16–18 h). The cells were washed twice with RPMI 1640, replenished with complete medium containing test articles (200 µL/well), and incubated further for 72 h, at which time half of the medium in each well (100 µL) was harvested for p24 ELISA, and 100 µL of fresh medium was added to replenish each well. Afterwards, cell culture supernatants were assayed for viral levels at the indicated time in a similar fashion.

### Inhibition of cell-to-cell HIV-1 infection in vitro following treatment with SAHA

PBMCs at 2.5×10^6^ cells/mL were cultured in complete medium for 48 h at 37°C in 5% CO_2_. Cells were then collected, washed, counted, suspended in fresh RPMI 1640 medium at 2.5×10^6^ cells/mL, and infected overnight with HIV-1_LAI_ (150–250 TCID_50_/mL). On the next day, cells were washed 3 times with complete medium, counted, dispensed (200 µL) onto 96-well cell culture plates at 150,000 cells/well, and grown in complete medium for 3 days, at which time 100 µL from each well was removed for p24 ELISA and 100 µL of fresh medium was added to replenish each well. After 7 days, all medium was harvested and cells were washed 3 times with complete medium. Jurkat T cells were then added to the wells (50,000 cells per well) in complete medium, half of which were also given SAHA at 100 nM, and serially diluted hLL2-(T20)_4_, T20, hLL2 and cP4/D10 were added to designated wells. Every third day, after removing 100 µL from each well for p24-ELISA, 100 µL of fresh medium were added to replenish each well and this operation was repeated over a period of 4 weeks.

### In vivo stability in mice

Naïve female SCID mice (16 to 22 g, N = 11) purchased from Taconic Farms (Germantown, NY) were housed in Thoren units with food and water *ad libitum*. Animals were injected s.c. with hLL2-(T20)_4_ (100 µg; 500 pmol) and anesthetized with ketamine/xylazine prior to collection of blood samples at 0.5, 6, 24 and 72 h from 2, 3, 3, and 3 mice, respectively, and stored at −70°C until analyzed by ELISA. A parallel study was performed with hLL2 IgG (75 µg; 500 pmol) in the same fashion. The serum samples from mice injected with hLL2-(T20)_4_ were examined by two different ELISAs, one designed to quantify only the intact hLL2-(T20)_4_ and the other to quantify all hLL2-containing species, with or without the linked T20. For quantification of hLL2-(T20)_4_, plates were coated with F(ab′)_2_-specific, goat anti-human IgG, and the captured antibodies probed with a mouse anti-DDD2 mAb (5E3) developed in-house, followed by HRP-conjugated goat-anti-mouse IgG. For measuring all hLL2-containing species, plates were coated with AffiniPure Goat Anti-Human IgG, F(ab′)_2_ Fragment Specific (Jackson ImmunoReserach, West Grove, PA) and the captured antibodies were probed with a rat anti-idiotype mAb to hLL2 (WN; Immunomedics, Inc.), followed by Peroxidase AffiniPure F(ab′)_2_ Frag Goat anti-Rat IgG, FcR Frag Spec (Jackson ImmunoReserach). The second assay was also used for measuring the serum levels of hLL2. The animal study was approved by the Institutional Animal Care and Use Committee (IACUC) of the Center for Molecular Medicine and Immunology (Morris Plains, NJ) in compliance with NCI guidelines.

### Statistical analysis

Statistical comparisons between the groups of data obtained with different compounds were performed using the nonparametric Mann-Whitney U and Kruska Wallis tests. A one-way ANOVA nonparametric test was performed using GraphPad Prism version 4.0a for Macintosh, or OS 9 Apple GraphicPad Software (San Diego, CA), and was used for comparisons of HIV-1 isolation and p24 antigen positivity between the study groups. A two-tailed *t*-test was used for comparisons between mouse serum concentrations of the different agents after analyzing the data with an F-Test (Microsoft Excel 2003) for possible difference in variance. *P*-values less than 0.05 were considered statistically significant.

## Supporting Information

Figure S1 **LC-MS analysis of DDD2-T20.** Electrospray ionization time of flight (ESI-TOF) liquid chromatography/mass spectrometry (LC-MS) was performed with a 1200-series HPLC coupled with a 6210 TOF MS (Agilent Technologies, Santa Clara, CA). The DDD2-T20 was reduced with 50 mM tris(2-carboxyethyl)phosphine for 30 min and resolved by reversed phase HPLC (RP-HPLC), using a 20-min gradient of 30–80% acetonitrile in 0.1% aqueous formic acid with a Jupiter C4 5 µ column (Phenomenex, Torrance, CA). For the TOF MS, the capillary and fragmentor voltages were set to 5000 and 200 V, respectively. The observed mass (11824.53 Da) closely matched (26 ppm) the calculated mass (11824.23) of the deduced amino acid sequence.
(PPTX)

Figure S2 **Size Exclusion HPLC analysis of IgG-(T20)_4_ constructs.** The hLL2-(T20)_4_ and cP4/D10-(T20)_4_ each resolved as a single protein peak at retention times of 7.85 min and 8.55 min, respectively, which were shorter than that of the hLL2-IgG-AD2 (8.33 min) and cP4/D10-IgG-AD2 (8.86 min), respectively, and consistent with their molecular size.
(PPT)

Figure S3 **Antibody binding measured by ELISA.** (A) P4/D10 and cP4/D10 showed similar binding avidity to recombinant gp160. (B) Comparative binding to a synthetic V3 peptide representing the third variable loop of HIV-1 gp120 outer envelope protein. Replicate samples were washed with 8 M urea or saline. For both P4/D10 and cP4/D10, similar antibody titer was measured with 8 M urea and saline, giving an avidity index of 0.98, suggesting a similarly strong binding avidity for each antibody.
(PPTX)

Table S1 Comparative potencies of anti-HIV fusion inhibitors.
(DOC)

Methods S1 **Methods are provided for: chimerization of P4/D10; construction of expression vectors for cP4/D10 and cP4/D10-AD2; expression and purification of chimeric P4/D10 and AD2-containing Abs; cloning, expression and purification of DDD2-T20; generation of IgG-(T20)_4_ constructs with DNL; antibody-binding ELISA.**
(DOC)
