# Supplementary material for: A Novel Class of Anti-HIV Agents with Multiple Copies of Enfuvirtide Enhances Inhibition of Viral Replication and Cellular Transmission In Vitro
Source: PLoS One. 2012 Jul 23;7(7):e41235. doi: 10.1371/journal.pone.0041235 (PMC3402531; doi:10.1371/journal.pone.0041235)
Supplement: Figure S1 — LC-MS analysis of DDD2-T20. Electrospray ionization time of flight (ESI-TOF) liquid chromatography/mass spectrometry (LC-MS) was performed with a 1200-series HPLC coupled with a 6210 TOF MS (Agilent Technologies, Santa Clara, CA). The DDD2-T20 was reduced with 50 mM tris(2-carboxyethyl)phosphine for 30 min and resolved by reversed phase HPLC (RP-HPLC), using a 20-min gradient of 30–80% acetonitrile in 0.1% aqueous formic acid with a Jupiter C4 5 µ column (Phenomenex, Torrance, CA). For the TOF MS, the capillary and fragmentor voltages were set to 5000 and 200 V, respectively. The observed mass (11824.53 Da) closely matched (26 ppm) the calculated mass (11824.23) of the deduced amino acid sequence. (PPTX) [file pone.0041235.s001.pptx]

## Slide 1
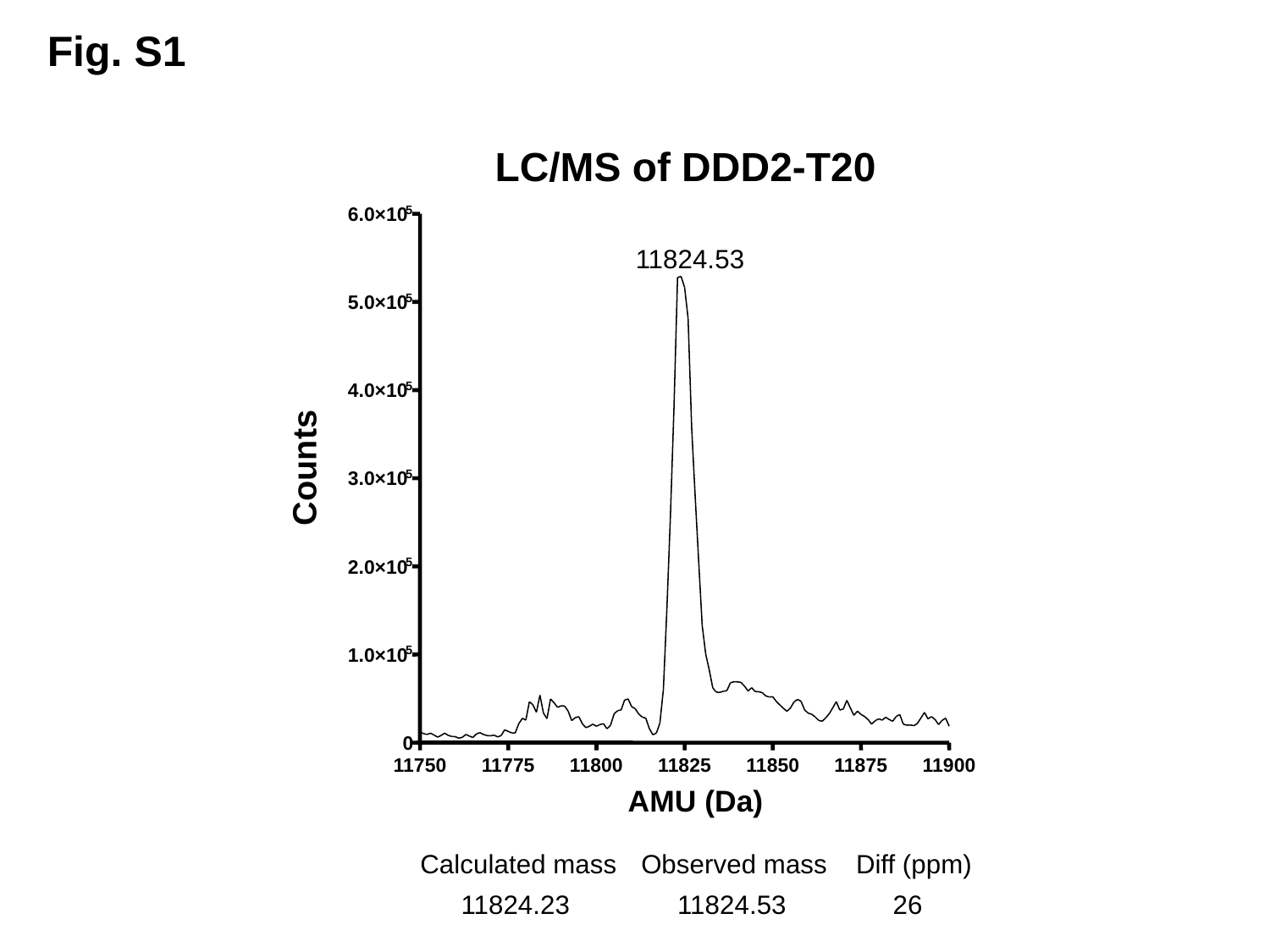

Fig. S1
LC/MS of DDD2-T20
6.0×10
5
11824.53
5.0×10
5
4.0×10
5
3.0×10
5
2.0×10
5
1.0×10
5
0
11750
11775
11800
11825
11850
11875
11900
Calculated mass
Counts
AMU (Da)
Observed mass
Diff (ppm)
11824.23
11824.53
26

## Slide 2
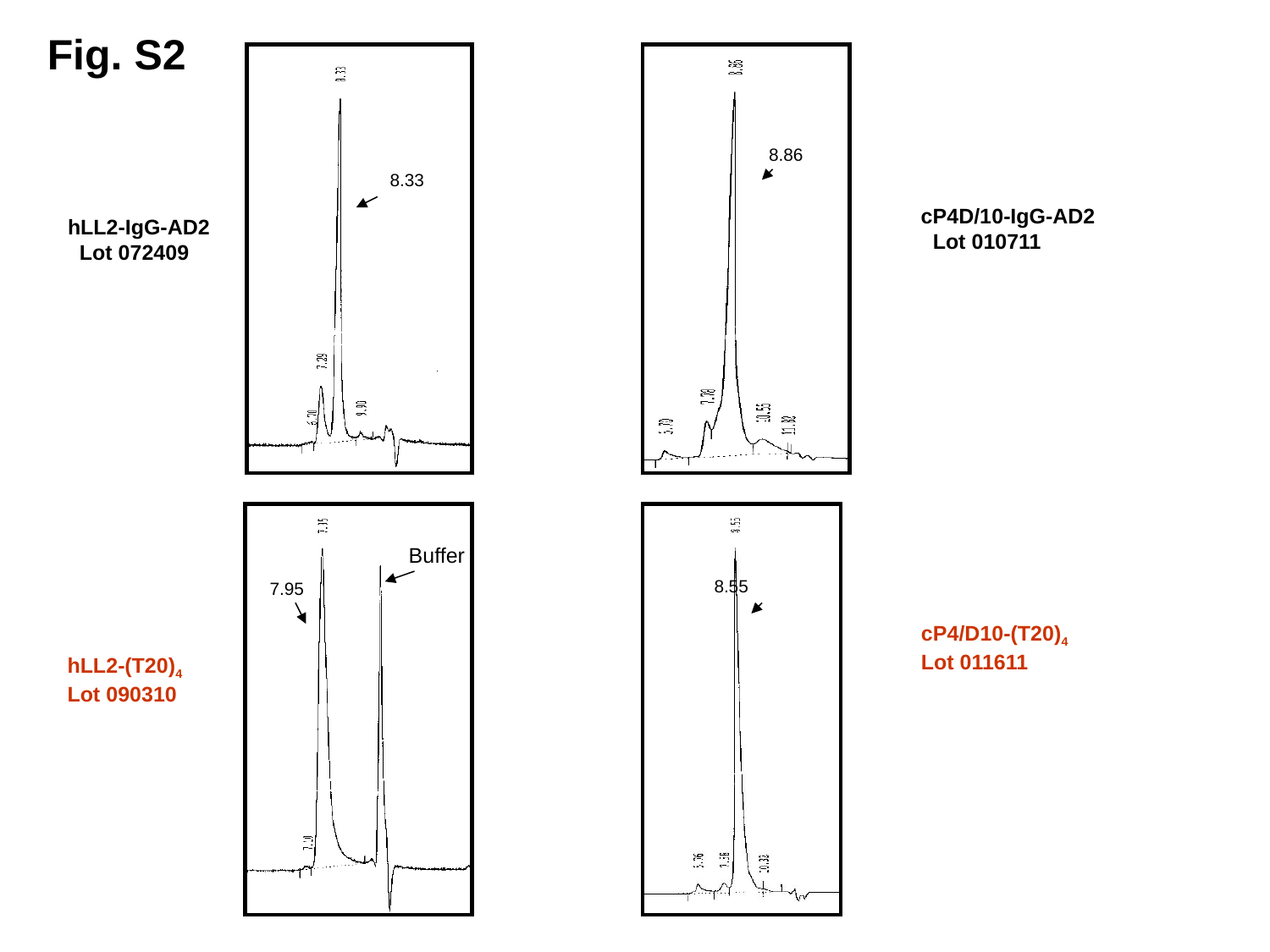

Fig. S2
8.86
8.33
cP4D/10-IgG-AD2
 Lot 010711
hLL2-IgG-AD2
 Lot 072409
Buffer
8.55
8.55
7.95
cP4/D10-(T20)4
Lot 011611
hLL2-(T20)4
Lot 090310

## Slide 3
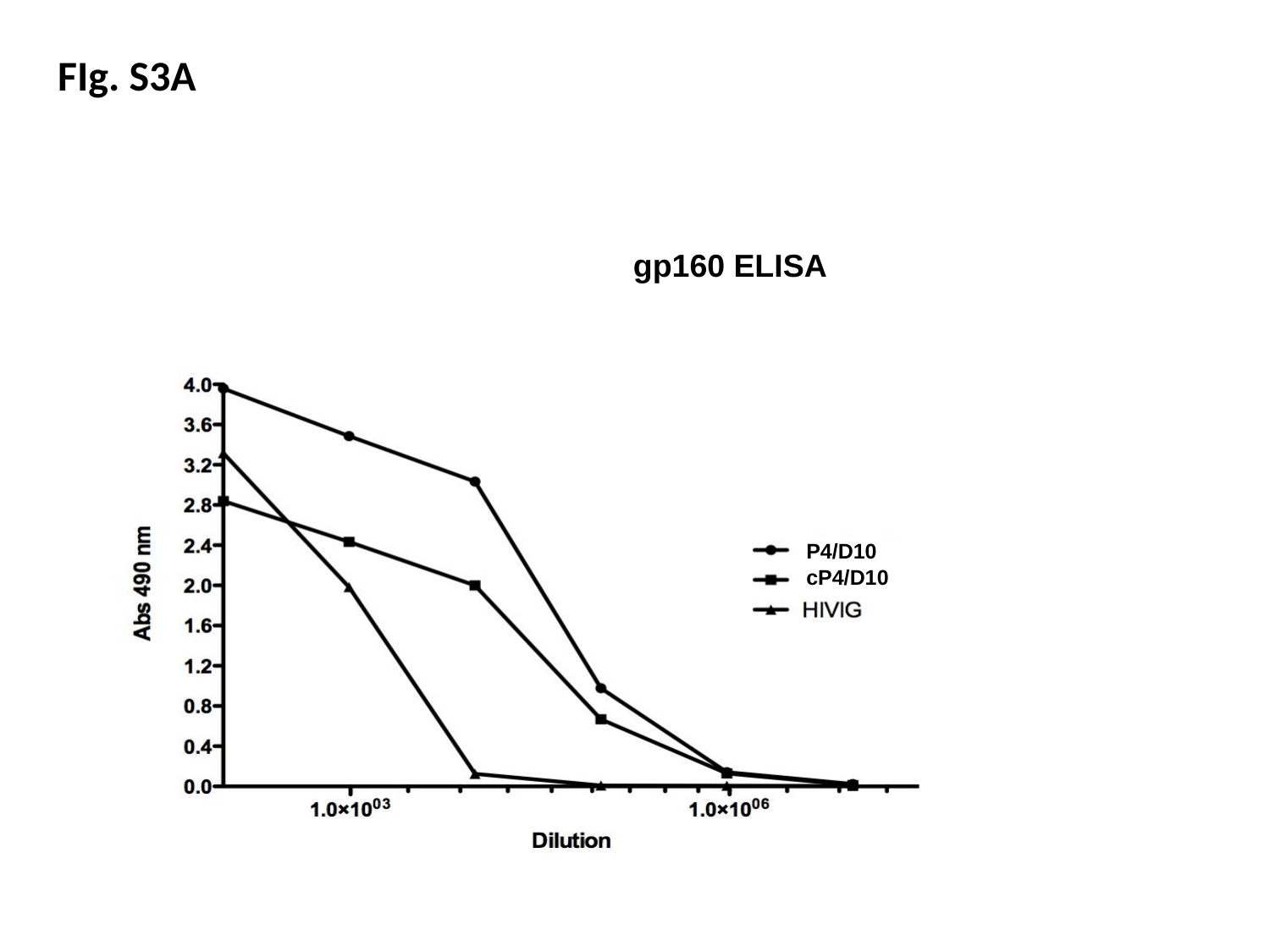

FIg. S3A
gp160 ELISA
P4/D10
cP4/D10

## Slide 4
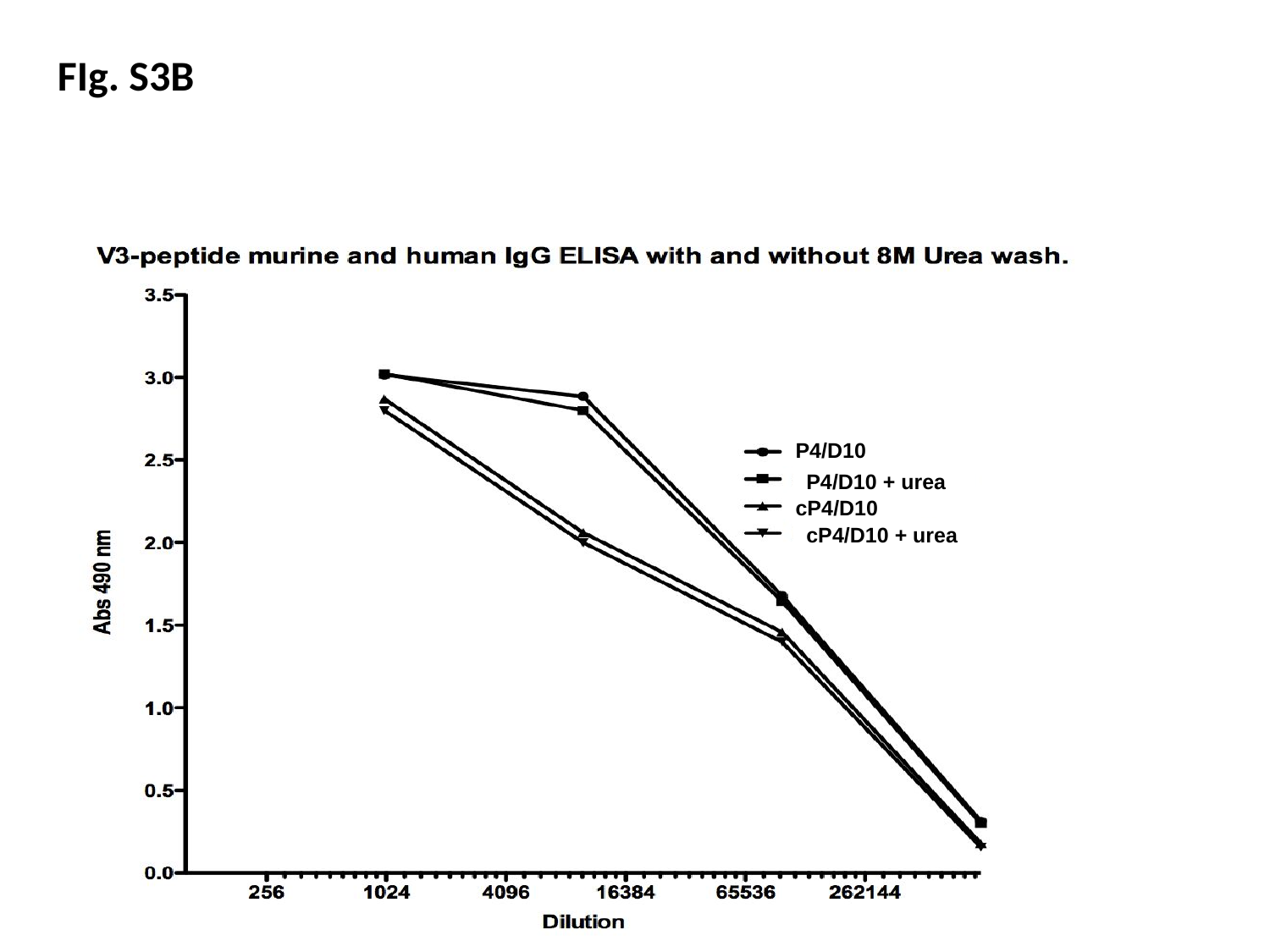

FIg. S3B
P4/D10
P4/D10 + urea
cP4/D10
cP4/D10 + urea
