# Supplementary figures and images for: A Novel Class of Anti-HIV Agents with Multiple Copies of Enfuvirtide Enhances Inhibition of Viral Replication and Cellular Transmission In Vitro
Source: PLoS One. 2012 Jul 23;7(7):e41235. doi: 10.1371/journal.pone.0041235 (PMC3402531; doi:10.1371/journal.pone.0041235)

## Slide 1
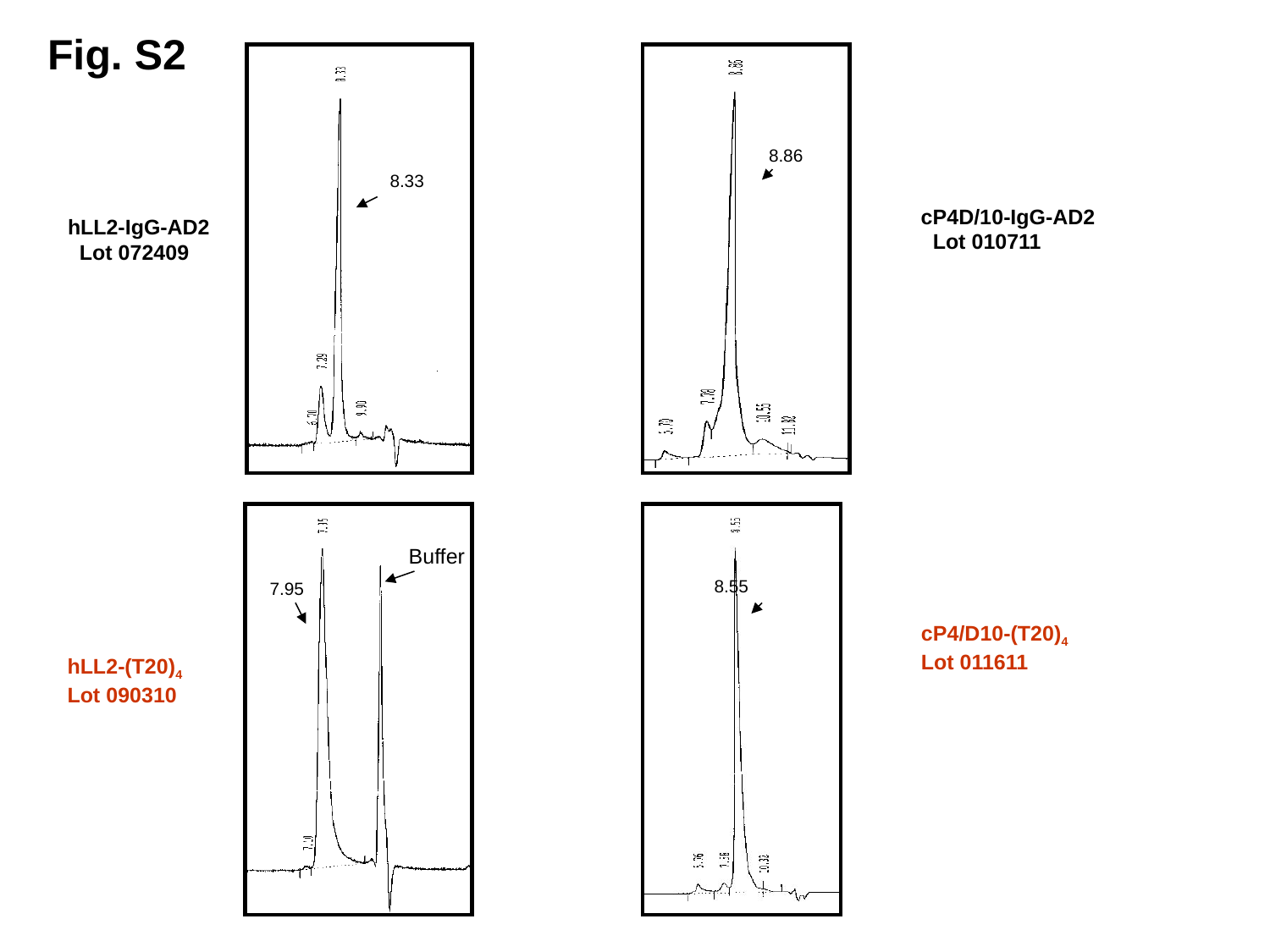

Fig. S2
8.86
8.33
cP4D/10-IgG-AD2
 Lot 010711
hLL2-IgG-AD2
 Lot 072409
Buffer
8.55
8.55
7.95
cP4/D10-(T20)4
Lot 011611
hLL2-(T20)4
Lot 090310

Supplement: Figure S2 — Size Exclusion HPLC analysis of IgG-(T20)4 constructs. The hLL2-(T20)4 and cP4/D10-(T20)4 each resolved as a single protein peak at retention times of 7.85 min and 8.55 min, respectively, which were shorter than that of the hLL2-IgG-AD2 (8.33 min) and cP4/D10-IgG-AD2 (8.86 min), respectively, and consistent with their molecular size. (PPT) [file pone.0041235.s002.ppt]

## Slide 1
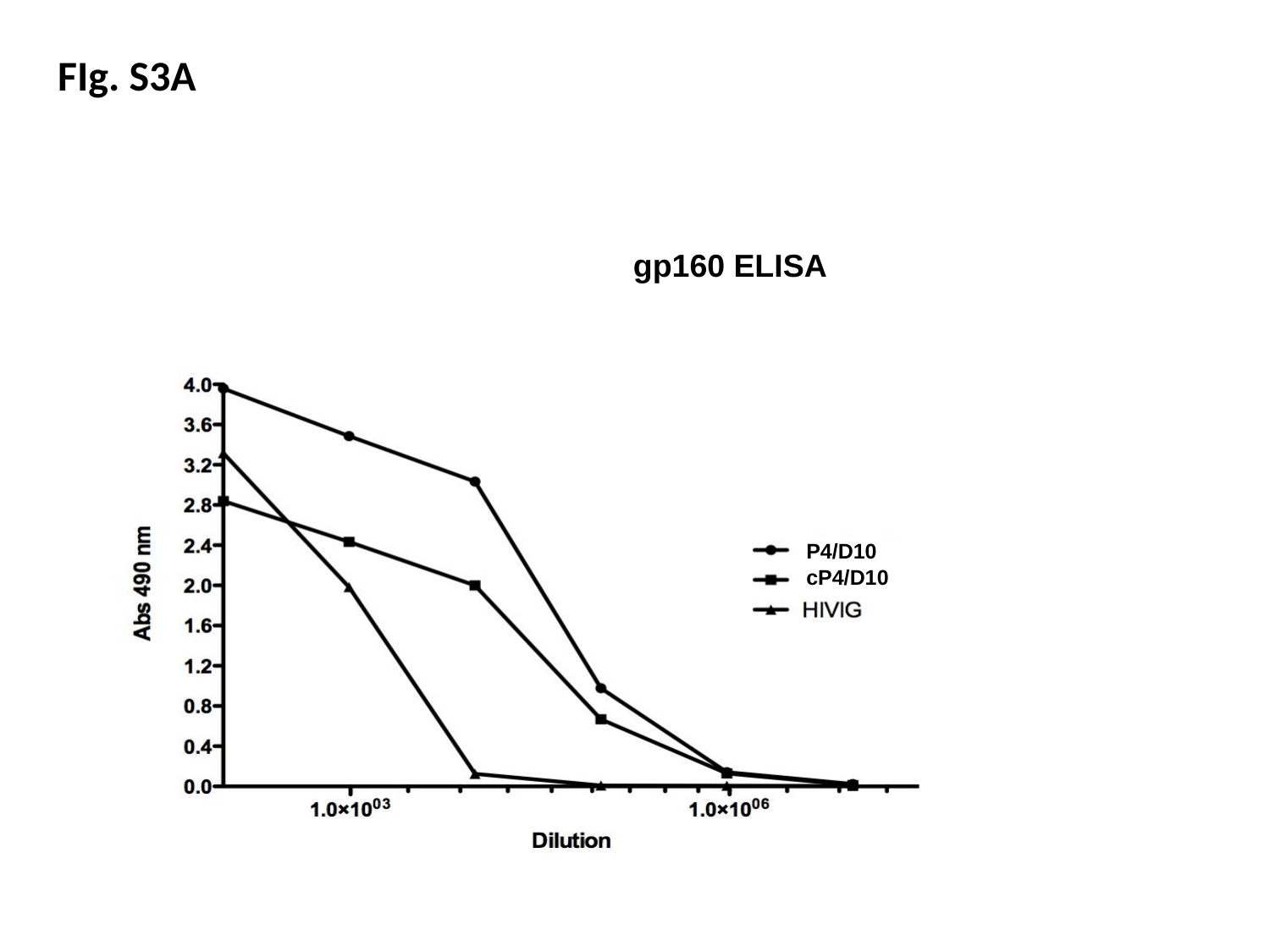

FIg. S3A
gp160 ELISA
P4/D10
cP4/D10

## Slide 2
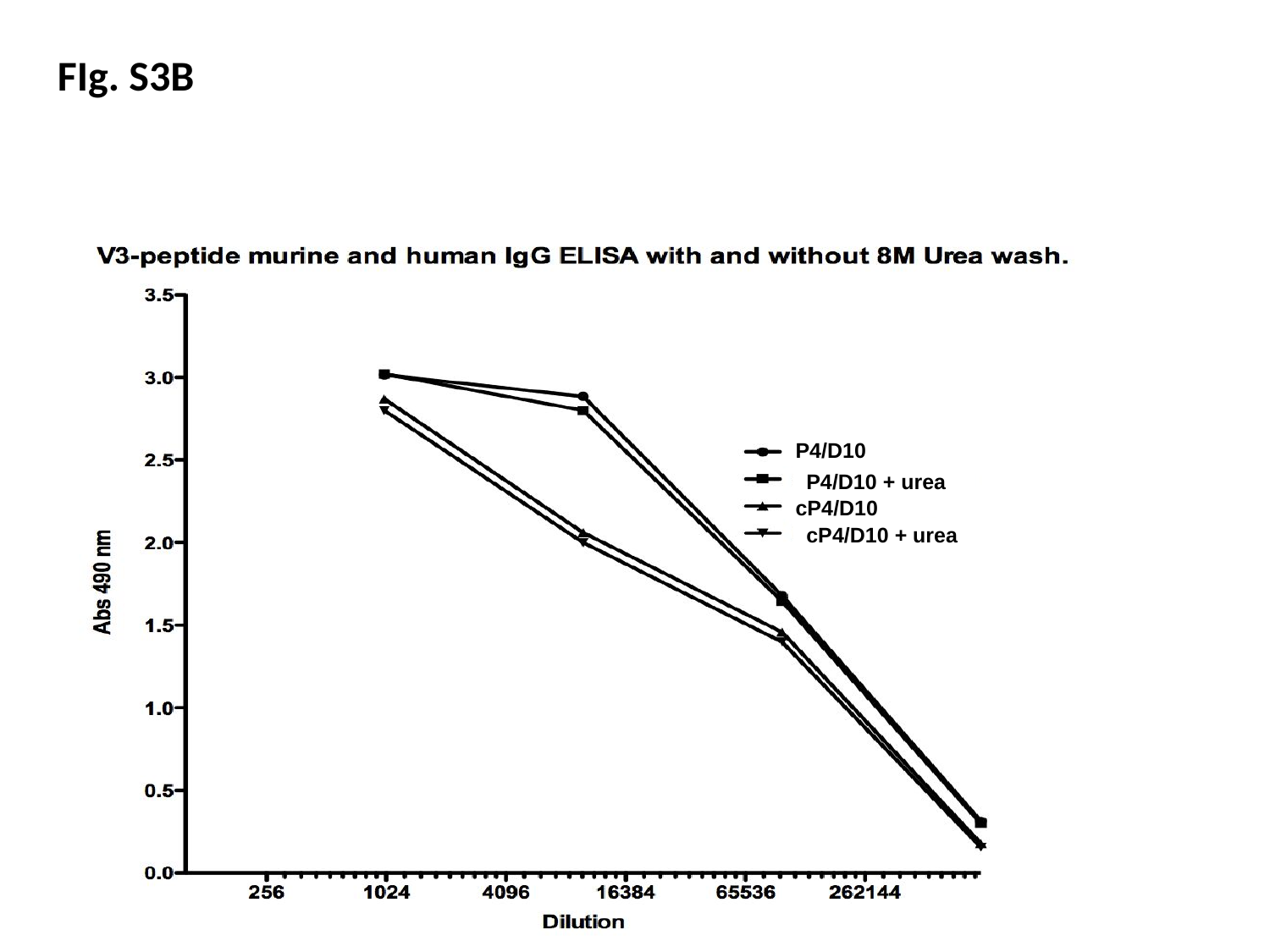

FIg. S3B
P4/D10
P4/D10 + urea
cP4/D10
cP4/D10 + urea

Supplement: Figure S3 — Antibody binding measured by ELISA. (A) P4/D10 and cP4/D10 showed similar binding avidity to recombinant gp160. (B) Comparative binding to a synthetic V3 peptide representing the third variable loop of HIV-1 gp120 outer envelope protein. Replicate samples were washed with 8 M urea or saline. For both P4/D10 and cP4/D10, similar antibody titer was measured with 8 M urea and saline, giving an avidity index of 0.98, suggesting a similarly strong binding avidity for each antibody. (PPTX) [file pone.0041235.s003.pptx]
