# Supplementary material for: A Novel Class of Anti-HIV Agents with Multiple Copies of Enfuvirtide Enhances Inhibition of Viral Replication and Cellular Transmission In Vitro
Source: PLoS One. 2012 Jul 23;7(7):e41235. doi: 10.1371/journal.pone.0041235 (PMC3402531; doi:10.1371/journal.pone.0041235)
Supplement: Table S1 — Comparative potencies of anti-HIV fusion inhibitors. (DOC) [file pone.0041235.s004.doc]

| Table S1.  **Comparative potencies of anti-HIV fusion inhibitors** | | | |
| --- | --- | --- | --- |
| HIV-fusion inhibitor | # of T20 or C34 | Anti-HIV potency | |
|  |  | EC_50_ | EC_90_ |
|  |  | (nM) | (nM) |
| T20 | 1 | 1 to 2 | ~10 |
| EP40111 | 1 (T20) | 100 | NA |
| Fc-T20 | 2 | 5 | NA |
| h734-(T20)_4_ | 4 | ~0.1 | ~0.6 |
| C34 | 1 | 0.6 | 2.8 |
| HSA-C34 (PC-1505) | 1 | 1.8 | 13.5 |
| All data shown were determined with a PBMC/p24-based assay. Potency is dependent in part on the specific HIV-strains and clades tested. EC_50_ values for T20 and h734-(T20)_4_ were based on the does-response curves shown in Fig. 2. EP40111 was made by site-specific conjugation of T20 to an anti-thrombin binding pentasaccharide via a PEG-12 linker (Huet et al., 2010, Antimicrob Agents Chemother 54:134-42). Data for Fc-T20 was as reported by Syntonix. C34 is a T20 analog (Stoddart et al., 2008, J Biol Chem 283:34045-52). PC-1505 is a T20 analog made by site-specific conjugation of maleimido-C34 to HSA (at Cys34). | | | |
