# Supplementary material for: A Novel Class of Anti-HIV Agents with Multiple Copies of Enfuvirtide Enhances Inhibition of Viral Replication and Cellular Transmission In Vitro
Source: PLoS One. 2012 Jul 23;7(7):e41235. doi: 10.1371/journal.pone.0041235 (PMC3402531; doi:10.1371/journal.pone.0041235)
Supplement: Methods S1 — Methods are provided for: chimerization of P4/D10; construction of expression vectors for cP4/D10 and cP4/D10-AD2; expression and purification of chimeric P4/D10 and AD2-containing Abs; cloning, expression and purification of DDD2-T20; generation of IgG-(T20)4 constructs with DNL; antibody-binding ELISA. (DOC) [file pone.0041235.s005.doc]

**Chimerization of P4/D10**

**RNA isolation, cDNA synthesis, and amplification**

Total RNA was prepared from 1x10^8^ cells of a P4/D10-expressing cell line using a Qiagen Rneasy Mini kit (Qiagen, Valencia, CA). The mRNA was further isolated from the total RNA using an Oligotex mRNA maxi kit (Qiagen). cDNA was synthesized from 2 μg of mRNA in one-step RT-PCR using gene-specific primers (Qiagen).

The V_k_ sequence was amplified using the primers:

*CK3’-BH* (5’-GCCGGATCCTCACTGGATGGTGGGAAGATGGATACA-3’) *VK1BACK* (5’-GACATTCAGCTGACCCAGTCTCCA-3’)

The V_H_ sequence was amplified using the primers:

*CH1C* (5’-AGCTGGGAAGGTGTGCAC-3’), anneals to the CH1 of murine IgG *VH1BACK* (5’-AGGTSMARCTGCAGSAGTCWGG-3’) [1-3].

The sequences of V_k_ and V_H_ were determined by DNA sequencing.

h

# Construction of expression vectors for cP4/D10 and cP4/D10-AD2

The cDNA sequences of V_k_ and V_H_ were synthesized by Genscript (Piscataway, NJ) with the addition of restriction endonuclease sites subsequently used for cloning. The V_k_ and V_H_ domains of P4/D10 were subcloned into the dicistronic mammalian vector pdHL2, resulting in an expression vector for chimeric P4/D10, designated *cP4/D10-pdHL2*. To construct the *cP4/D10-AD2* expression vector, a fragment encoding the genomic sequence for the heavy chain constant domains (C_H_1–C_H_2-C_H_3) was excised from *cP4/D10-pdHL2* and replaced with a fragment comprising C_H_1-C_H_2-C_H_3-AD2, which was excised from an existing *IgG-AD2*-*pdHL2* vector.

**Expression and purification of chimeric P4/D10 and AD2-containing Abs**

Transfection, selection, and purification of cP4/D10 were as described previously [4]. Briefly, 30 μg of *cP4/D10-pdHL2* (or *AD2-cP4/D10-pdHL2*) was linearized by digestion with Sal I restriction enzyme and used to transfect SpESFX-10 cells by electroporation [5]. After 2 days, 0.2 μM methotrexate (MTX) was added for selection. MTX-resistant clones were screened for mAb secretion by ELISA and higher producing clones were expanded for further evaluation. For production of antibody, cells were cultured in roller bottles with serum-free H-SFM media containing MTX. Antibody was purified from the culture supernatant fluid by Protein A affinity chromatography using MAb-Select resin. Protein concentration was determined by SE-HPLC and OD_280_.

**Cloning, expression and purification of DDD2-T20**

cDNA encoding DDD2-T20 was synthesized and cloned into the *E. coli* expression vector pET26b, in frame with the *PelB* signal peptide and 6-His tag (Novagen). Chemically competent Rosetta (DE3)pLysS *E. coli* were transformed with DDD2-T20-pET26b and cultures grown in 2xYT broth were induced with 0.4 mM IPTG at OD_600_=0.8 at 30°C for 3 h. The cell pellet was frozen at -70°C, thawed at room temperature, and resuspended in binding buffer (10 mM imidazole, 300 mM NaCl, 50 mM NaH_2_PO_4_, pH 8.0). The suspension was homogenized on ice using a tissue homogenizer and clarified by centrifugation at 17,000 rpm for 15 minutes. The DDD2-T20 was purified by immobilized metal affinity chromatography using His-Select resin (Sigma). The column was washed with buffer containing 30 mM imidazole prior to elution with 250 mM imidazole.

**Generation of IgG-(T20)_4_ constructs with DNL**

Three IgG-(T20)_4_ conjugates, cP4/D10-(T20)_4_, hLL2-(T20)_4_ and h734-(T20)_4_, were synthesized using the Dock-and-Method as described previously [4]. The respective IgG-AD2 modules were mixed with excess DDD2-T20 and held at room temperature overnight in the presence of 2 mM reduced glutathione and 1 mM EDTA. Oxidized glutathione was added to 4 mM and the solution was again held overnight at room temperature. The IgG-(T20)_4_ were purified by sequential affinity chromatography steps using Protein A and His-Select affinity columns. Each purified IgG-(T20)_4_ conjugate resolved as a single protein peak by SE-HPLC with retention times consistent with their molecular size (~195 kDa) (Fig S2).

As an example, a 5-mg-scale preparation of hLL2-(T20)_4_ was produced as follows. C_H_3-AD2-IgG-hLL2 (5.2 mg in 4 mL of 0.1 M Tris, 0.1 M citrate, pH 7.5) was combined with a two-fold molar excess of DDD2-T20 (0.52 mg in 2 mL of 250 mM imidazole, 150 mM NaCl, 50 mM NaH_2_PO_4_, pH 8.0). Reduced glutathione and EDTA were added to final concentrations of 2 mM and 1 mM, respectively, and the mixture was reduced for 16 h at room temperature. Oxidized glutathione was then added to 4 mM final concentration and the mixture was held for an additional 20 h at room temperature, before application on a 1-mL Mab-Select chromatography column. The protein A column was washed to baseline with PBS and the conjugate was eluted with 0.1 M glycine, pH 2.5. The eluate was adjusted to 10 mM imidazole, 300 mM NaCl, 50 mM NaH_2_PO_4_, pH 8.0 and applied to a 1-mL His-Select IMAC column, which was washed with 30 mM imidazole buffer. A total of 4.5 mg of highly-purified hLL2-(T20)_4_ was eluted with 250 mM imidazole buffer.

**Antibody-binding ELISA**

Nunc, 96-microwell plates were coated overnight at room temperature with recombinant gp160 antigen (0.5 μg/mL, Protein Sciences, Meriden, CT) or a synthetic V3 peptide (10 μg/mL, Ferring, Malmo, Sweden) representing the third variable loop of HIV-1 gp120 outer envelope protein. Antibodies were serially diluted in 0.5% BSA, 0.05% Tween 20, PBS and incubated in the coated wells at 37°C for 60 min. Binding was detected with HRP-labeled anti-mouse or anti-human IgG conjugate (1/30,000; BioRad, Richmond; CA) and the signal was developed with O-phenylene diamine substrate.

An additional washing step was added for the avidity assay [6]. One set of antibody-bound wells was washed with 8M Urea/PBS buffer for 5 min at room temperature prior to detection and signal development. The avidity index (AI) is defined as the ratio of the titer obtained from the 8M urea-washed to the saline-washed replicates.

**References**

1. Orlandi R, Gussow DH, Jones PT, Winter G (1989) Cloning immunoglobulin variable domains for expression by the polymerase chain reaction. Proc Natl Acad Sci USA 86: 3833-3837.

2. Qu Z, Losman MJ, Eliassen KC, Goldenberg DM, Leung SO (1999) Humanization of Immu31, an α-Fetoprotein-specific Antibody. Clin Cancer Res 5(10 Suppl): 3095s-3100.

3. Leung SO, Dion AS, Pellegrini MC, Goldenberg DM, Hansen HJ (1993) An extended primer set for PCR amplification of murine k variable regions. Biotechniques 15: 286-292.

4. Rossi EA, Goldenberg DM, Cardillo TM, Stein R, Wang Y, et al. (2008) Novel designs of multivalent anti-CD20 humanized antibodies as improved lymphoma therapeutics. Cancer Res 68: 8384-8392.

5. Rossi DL, Rossi EA, Goldenberg DM, Chang CH (2011) A new mammalian host cell with enhanced survival enables completely serum-free development of high-level protein production cell lines. Biotechnol Prog 27: 766-775.

6. Korhonen MH, Brunstein J, Haario H, Katnikov A, Rescaldani R, et al. (1999) A new method with general diagnostic utility for the calculation of immunoglobulin G avidity. Clin Diagnost Lab Immunol 6: 725-728.
